# Metal Marvels: Revolutionizing Endodontic Restoration With a Novel Endocrown Approach

**DOI:** 10.7759/cureus.55319

**Published:** 2024-03-01

**Authors:** Paridhi Agrawal, Aishwarya Rathod, Priyanka Jaiswal, Deepika Masurkar, Manoj Chandak, Aditya Patel, Jay Bhopatkar

**Affiliations:** 1 Department of Conservative Dentistry and Endodontics, Sharad Pawar Dental College and Hospital, Datta Meghe Institute of Higher Education & Research, Wardha, IND; 2 Department of Periodontics, Sharad Pawar Dental College and Hospital, Datta Meghe Institute of Higher Education & Research, Wardha, IND

**Keywords:** crown, endodontic rehabilitation, base metal alloy, prosthesis, endocrown

## Abstract

This case report explores the innovative application of the endocrown technique for restoring a severely damaged mandibular molar (tooth #46) in a 28-year-old male patient. With a recent root canal treatment history, the patient presented with a dislodged prosthesis. Due to financial constraints, a base metal alloy was chosen for the endocrown restoration. The unique preparation process involved reducing the occlusal surface by 2 mm, creating a shoulder cervical margin, and preserving enamel walls. The endocrown, crafted from a base metal alloy, demonstrated a semi-conservative approach, providing cost-effectiveness and minimal tooth preparation. The case adheres to the 2013 CAse REport (CARE) guidelines. The discussion highlights the biomechanical benefits of the endocrown, emphasizing stress resistance, stability, and superior performance compared to traditional treatments. Materials like ceramic, resin nanoceramic, and polyetheretherketone are briefly discussed, focusing on the promising success rates of endocrowns, mainly through computer-aided designing/computer-aided manufacturing systems. The report provides valuable insights for clinicians considering this endocrown technique in reconstructing severely damaged molars and premolars.

## Introduction

The importance of post-endodontic restoration goes far beyond merely preserving and safeguarding the remaining tooth structure. Its significance lies in the restoration’s role in reinstating esthetics, form, and function in teeth undergoing endodontic treatment. The process plays a crucial part in conserving the tooth’s structural integrity and achieving mechanical stabilization of the tooth-restoration complex. This is accomplished through minimally invasive tooth preparations that prioritize maximal tissue preservation. By adopting such an approach, clinicians can effectively enhance the longevity of endodontically treated teeth, contributing to the overall durability and sustainability of the tooth-restoration complex [1].

Addressing the challenge of restoring severely damaged teeth in the coronal region is no small feat for practitioners. The longevity of such restorations depends on various factors, including occlusion, function, and esthetics. To meet this challenge, Bindl and Mormann introduced the concept of “endocrown,” a conservative remedial alternative designed for restoring nonvital teeth. The endocrown utilizes pulp chamber support to create a definitive onlay restoration meticulously crafted from ceramic material [2]. So, endocrowns are monolithic, single-piece restorations that repair the coronal area of a tooth that has undergone endodontic treatment, either completely or partially, that count on macromechanical retention, which is accomplished by securing the repair to the cavity’s edge as well as the interior section of the pulp chamber, and additionally on adhesive cementation for micromechanical retention [3].

Endodontically treated teeth differ significantly from their vital counterparts in terms of both structural and physical integrity. These teeth change due to the loss of tooth structure caused by factors such as caries or trauma, coupled with biomechanical alterations leading to reduced elasticity and increased brittleness. These changes make endodontically treated teeth more susceptible to fractures [4]. In response to these challenges, endocrowns made from ceramic or zirconia emerge as favorable alternatives to full-coverage restorations for nonvital posterior teeth. They are particularly indicated for teeth with minimal coronal architecture, where sufficient tooth support is available for robust adhesive cementation [5].

Although ceramic and zirconia crowns are effective, they can be relatively expensive, with ceramic requiring careful consideration of thickness for optimal strength. This case report introduces a distinctive approach - a case that employed an endocrown made from a base metal alloy. This innovative choice served as a semi-conservative treatment modality for post and core, offering cost-effectiveness, reduced clinical time, and minimal tooth preparation for an endodontically treated tooth. Importantly, the present case report adheres to the 2013 CAse REport (CARE) guidelines for reporting case reports [6].

## Case presentation

A 28-year-old male patient presented to the Department of Conservative Dentistry and Endodontics with the chief complaint of a dislodged prosthesis in the lower right posterior region of the jaw, a concern that has persisted for the past two months. The patient was in good health until two months ago, when the prosthesis in the lower right region got dislodged. The patient had undergone root canal treatment in the same tooth as the complaint tooth three months prior at a private dental clinic. The patient’s medical history did not contribute to the current issue.

Upon clinical and radiographic examination, a dislodged restoration was observed on tooth number 47. Tenderness on vertical and lateral percussion was not evident, and there were no signs of associated pain, swelling, or pus discharge. The patient maintained acceptable oral hygiene, and occlusion was found to be favorable. The radiographic assessment revealed a well-executed root canal filling, an absence of periapical lesions, and the dislodged coronal restoration on tooth 47.

Considering the thickness of the remaining walls and the amount of intact tooth structure, a decision was made to rehabilitate tooth 47 using an endocrown prosthesis. Although the initial prosthetic recommendation was a lithium disilicate ceramic crown, financial constraints led to the patient’s finalization of a metal crown as the chosen restorative option.

Endocrown preparation

Because of the unique characteristics of this restoration and its specific biomechanical requirements, the endocrown preparation differs from the conventional full crown. The principal objective was to accomplish a thorough occlusal surface height decrease of at least 2 mm in an axial direction, forming a cervical edge, often called a “cervical sidewalk,” in the shape of a butt joint.

**Figure 1:**
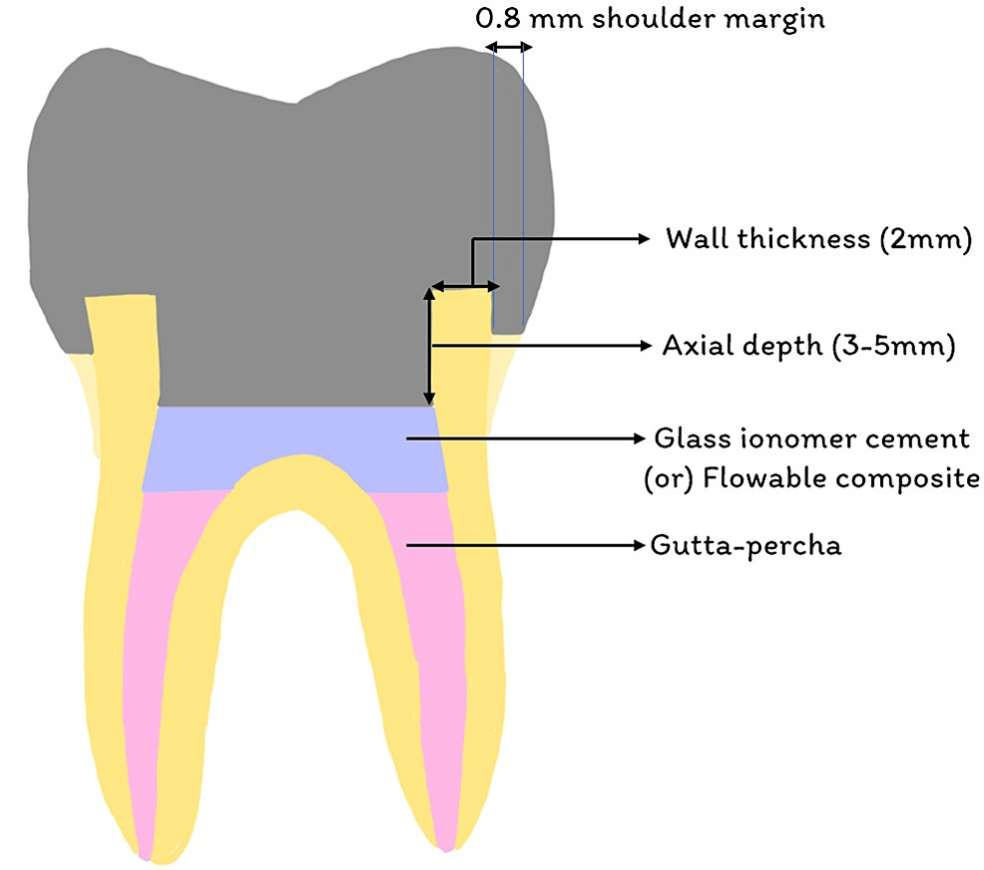
Schematic diagram of endocrown preparation Image credit: Paridhi Agrawal

Critical aspects of the preparation included ensuring that there was a supragingival cervical margin and that the enamel walls of thickness below 2 mm were eliminated. To avoid a staircase effect, any level differences between the areas of the cervical margin were connected by a slope not exceeding 60°. The chosen approach involved utilizing a cylindrical-conical diamond bur, which was placed parallel to the plane of occlusion, effectively reducing the occlusal surface while adhering to these specific guidelines. As shown in Figure 1, the tailored preparation technique was integral to optimizing the biomechanical performance and longevity of the endocrown restoration.

Following this, a diamond wheel bur regulated the reduction’s orientation and guaranteed a smooth surface. A conical-cylinder diamond bur with a total occlusal convergence of 7° was used to form a continuation between the endodontic access cavity and the chamber of the pulp. The preparation was done, ensuring no contact with the pulpal floor.

**Figure 2:**
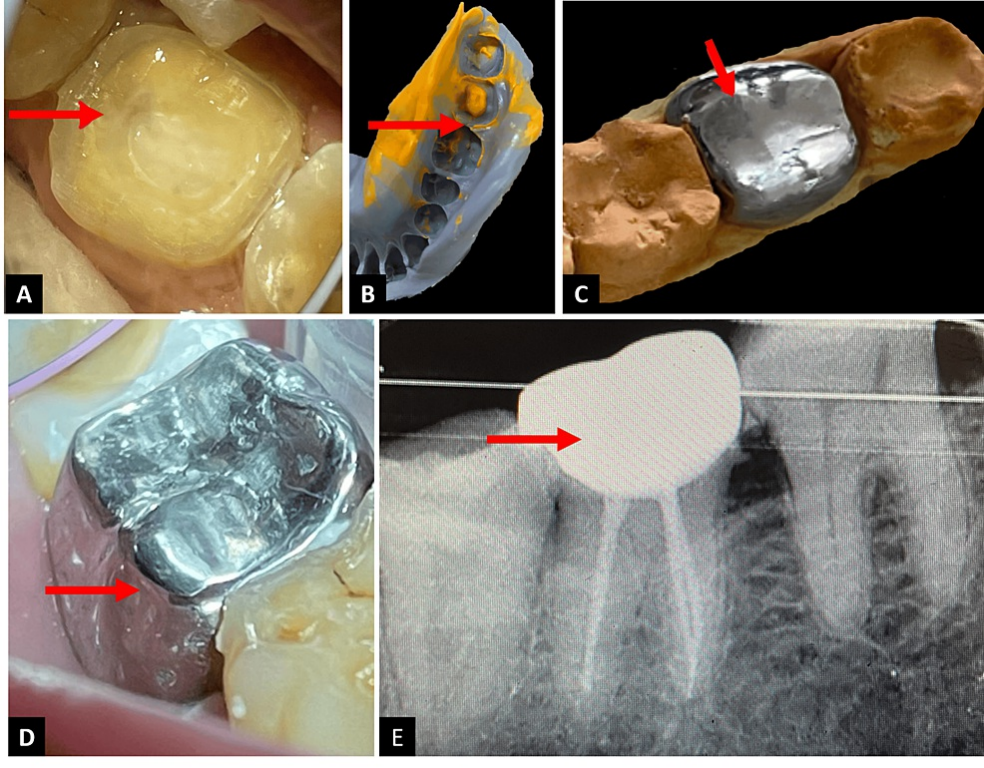
Endocrown with tooth number 47 (A) Endocrown preparation with tooth number 47. (B) Impression after endocrown preparation with tooth number 47. (C) Metal endocrown fabricated with tooth number 47. (D) Post-cementation clinical picture showing endocrown placement with tooth number 47. (E) Post-cementation radiograph showing endocrown placement with tooth number 47.

Vigilance was crucial because excessive tissue removal from the walls of the pulp chamber could impact the breadth and thickness of the enamel strip. The preparation was finished by covering the chamber of the pulp with glass ionomer cement (GIC; GC Gold Label, GC Corporation, Tokyo, Japan) to seal the canal opening, maintaining the cavity depth of at least 5 mm (Figure 2A).

After a thorough assessment of the entire cavity and interocclusal area, an impression was made using a single-step dual-viscosity impression technique with the addition of silicone impression material (Reprosil LV Tube, Aquasil Soft Putty, Dentsply Sirona Inc., Charlotte, North Carolina, USA), as shown in Figure 2B.

Following a meticulous inspection and evaluation of the impression quality, it was forwarded to the lab for additional processing. A temporary crown (Integrity Temporary Crown and Bridge Material, Dentsply Sirona Inc.) was created employing the block approach and sealed with a temporary cement (Integrity, TempGrip, Dentsply Sirona Inc.). Subsequently, a master cast was obtained, and a nickel-chromium base metal alloy endocrown was then fabricated in the laboratory (Figure 2C).

A try-in of the endocrown was then conducted, during which occlusion, internal, and proximal adjustments were checked. The intaglio surface of the endocrown was then sandblasted to improve the adhesion potential. The final endocrown was then securely bonded using glass ionomer type I cement (GC Gold Label, GC Corporation). The occlusion was thoroughly evaluated during this process. Excess cement was meticulously removed using an explorer to ensure optimal results, and dental floss was passed through proximal surfaces. The dental floss was then removed horizontally from the buccal cervical embrasure to clear off any loose GIC particles, ensuring a precise and well-finished restoration (Figure 2D and Figure 2E). The patient was then recalled for follow-up after one month, three months, six months, and nine months. Despite missing the scheduled follow-up appointments at one, three, six, and nine months, the patient expressed satisfaction with the provided treatment during a telephonic conversation.

## Discussion

Comprehensive management of extensively damaged molars poses a significant clinical challenge, necessitating meticulous planning for effective restorative interventions. To ensure sustained clinical viability and favorable outcomes, dental practitioners must judiciously evaluate their treatment options.

The endocrown emerges as a pertinent solution for diverse molar cases, particularly those characterized by small canals, calcified root canals, or clinically low crowns [7]. Nonetheless, caution is necessary in all cases, particularly where the pulpal chamber depth is less than 3 mm, adhesion cannot be assured, or the cervical edge is less than 2 mm throughout most of its circumference [8]. The efficacy of this method lies in its ability to facilitate straightforward impression capturing while preserving periodontal health [9]. Furthermore, the cohesiveness is heightened by the single interface of a one-piece restoration, optimizing its clinical utility [10].

During the preparatory phase, the objective is to furnish molars with a broad, robust surface capable of withstanding daily compressive forces [11]. By ensuring that the prepared surface and the occlusal plane are parallel, stress resistance on the central axis can be achieved [12]. Advancements in adhesive cementation technologies have obviated the need for macro-retentive crown preparation, alleviating stress on teeth endowed with endocrowns instead of those with prosthetic crowns [13]. The trapezoidal configuration in the mandibular molars and the triangular form in the maxillary molars of the pulpal chamber cavity contribute significantly to retention and stability, obviating the necessity for more preparation. The floor of the pulp with saddle form further augments strength, mitigating the vulnerability of root canals to drilling-induced fragility and averting stresses linked to post-usage [4].

Dartora et al. investigated the biomechanical performance of the endodontically treated teeth involving multiple endocrown extensions within the pulp chamber and stated that an extension of 5 mm demonstrates reduced magnitude and a more favorable pattern of stress distribution; a larger endocrown extension yields better mechanical performance [12], and so was seen in this case, where a 5 mm extension of endocrown in the pulp chamber was given to impart mechanical strength to the tooth.

According to Schultheis et al., the bilayer shape of the endocrown renders it a more dependable option for posterior load-bearing teeth, characterized by diminished fracture failure probability [13]. According to Biacchi et al., endocrowns can be used to maintain the biomechanical integrity of posterior nonvital teeth while still offering sufficient esthetics and function. They can also demonstrate resilience against the negative effects of the degeneration of the hybrid layer [4]. Comparative analyses, such as finite element assessments, indicate that teeth restored with endocrowns manifest heightened resilience against failure compared to those treated with traditional methodologies like posts and cores [14].

The potential superiority of endocrowns in terms of fracture strength and survival is highlighted by Sedrez-Porto et al.’s systematic review, which included in vitro trials of fracture strength and clinical trials on the survival of endocrowns in contrast to the standard procedures [15]. Darwish et al.’s experimentation underscores the positive outcomes associated with using resin nanoceramic endocrowns for maxillary premolars following endodontic treatment [16]. Zoidis et al. propose polyetheretherketone as a viable substructure for an endocrown restoration, emphasizing the need for additional long-term clinical data to substantiate their findings [17].

With reported promising success rates facilitated by computer-aided designing/computer-aided manufacturing (CAD/CAM) systems, endocrowns emerge as a reliable modality for the reconstruction of severely compromised molars and premolars, even in scenarios involving substantial coronal tissue loss or occlusal risk factors such as bruxism or unfavorable occlusal relationships [18,19]. With a follow-up period of three to 19 years, the reported success rate of molar endocrowns ranges between 72.73% and 99.57% [20].

Endocrowns are indicated when up to 50% of the coronal tooth structure is absent, providing complete occlusal coverage conservatively. They address challenges such as limited interocclusal space, significant loss of coronal dental structure, and issues like short or curved roots, calcified canals, and instrument breakage. Recommended for cases with insufficient clinical crown length, endocrowns overcome limitations in achieving a proper ferrule and offer an alternative in situations where intra-radicular posts may be restricted [21]. Because of the greater chance of tooth fracture and the poor retention of the restoration, the use of an endocrown may not be recommended in cases of significant dental tissue loss when the finish line of the endcrown is entirely below the cement-enamel junction after preparation. Furthermore, a full-coverage crown with or without a post is the recommended course of therapy when there is evidence of increased functional and lateral loads, as demonstrated by steep occlusal architecture, wear facets, or parafunction [21].

This approach to giving a metal endocrown exhibits strengths such as cost-effectiveness, conservation of tooth structure, stability, durability, and an efficient treatment process. The consideration of the patient’s financial constraints reflects a patient-centered approach. However, limitations arise in terms of aesthetics, as the metal prosthesis may not seamlessly integrate with natural teeth, potentially causing aesthetic concerns. Additionally, there is a risk of allergic reactions for some patients, and the use of CAD/CAM technology could have provided better adaptation.

## Conclusions

In light of existing evidence, the development of endocrowns presents itself as a reliable alternative for the restoration of moderately compromised non-vital posterior teeth. Particularly noteworthy is the prominence of metal endocrowns, which offer a cost-effective and conservative treatment avenue for teeth that have undergone root canal procedures. However, it is crucial to underscore that the establishment of long-term follow-up protocols and the conduct of prospective clinical studies are imperative. These endeavors are essential to ensure and substantiate the universal success and efficacy of metal endocrowns in diverse clinical scenarios.
